# Factors associated with delayed diagnosis of leprosy in an endemic area in Northeastern Brazil: a cross-sectional study

**DOI:** 10.1590/0102-311XEN113123

**Published:** 2024-01-08

**Authors:** Glicya Monaly Claudino dos Santos, Rachel L. Byrne, Ana Isabel Cubas-Atienzar, Victor Santana Santos

**Affiliations:** 1 Universidade Federal de Sergipe, Aracaju, Brasil.; 2 Liverpool School of Tropical Medicine, Liverpool, U.K.; 3 Universidade Federal de Sergipe, Lagarto, Brasil.

**Keywords:** Leprosy, Delayed Diagnosis, Disability Evaluation, Hanseníase, Diagnóstico Tardio, Avaliação da Deficiência, Lepra, Diagnóstico Tardío, Evaluación de la Discapacidad

## Abstract

This study aimed to investigate the factors related to the individual and the health system that contribute to delayed diagnosis of leprosy in an endemic area in the Northeastern Brazil. This is a cross-sectional study of 120 individuals with leprosy. Demographic and clinical data and information on the factors related to the individual and the health system that contribute to delayed diagnosis of leprosy were obtained. Delayed diagnosis in months was estimated for each participant by interviews. A multivariate Poisson’s regression analysis was performed between the outcome and the independent variables. The median delay in the diagnosis of leprosy was 10.5 (4.0-24.0) months. Approximately 12.6% of participants had grade 2 disability (G2D) at the time of diagnosis. In the multivariate Poisson regression analysis, males, older age, low schooling level, residing in urban areas, multibacellar or tuberculoid leprosy, not seeking healthcare immediately after symptom onset, suspected leprosy, excessive referrals, and the need for three or more consultations to confirm the diagnosis were associated with longer diagnostic delay. This study found a significant delay in the diagnosis of leprosy in Arapiraca, Northeastern Brazil, which may explain the continuously high rate of G2D among new cases. Factors related to the individual and the health system were associated with longer diagnostic delay. Interventions to raise awareness of the disease among the general population and strengthen primary health care are urgently needed.

## Resumo

Neste estudo objetivou-se investigar os fatores relacionados ao indivíduo e ao sistema de saúde que contribuem para o atraso no diagnóstico da hanseníase em uma área endêmica no Nordeste do Brasil. Trata-se de um estudo transversal que incluiu 120 pacientes com hanseníase. Foram obtidos dados demográficos, clínicos e informações sobre fatores relacionados ao indivíduo e ao sistema de saúde que contribuem para o atraso no diagnóstico da hanseníase. O atraso do diagnóstico em meses foi estimado para cada participante por meio de entrevistas. Foi realizada uma análise multivariada por regressão de Poisson entre o resultado e as variáveis independentes. A mediana de atraso no diagnóstico da hanseníase foi de 10,5 (4,0-24,0) meses. Aproximadamente 12,6% dos participantes apresentavam grau de incapacidade física 2 (GIF 2) no momento do diagnóstico. Na análise multivariada por regressão de Poisson, homens, idosos, baixa escolaridade, residir em área urbana, hanseníase multibacilar, hanseníase tuberculóide, não procurar atendimento imediatamente após perceber os primeiros sintomas, suspeita de hanseníase, encaminhamentos excessivos e três ou mais consultas necessárias para confirmação diagnóstica associaram-se ao maior atraso diagnóstico. Este estudo encontrou um atraso significativo no diagnóstico da hanseníase em Arapiraca, Nordeste do Brasil, o que pode explicar a taxa continuamente alta de GIF 2 entre os casos novos. Fatores relacionados ao indivíduo e ao sistema de saúde foram associados ao maior atraso no diagnóstico. Intervenções para aumentar a conscientização sobre a doença entre a população geral e fortalecer a atenção primária à saúde são urgentemente necessárias.

## Resumen

El objetivo de este estudio fue investigar los factores relacionados con el individuo y el sistema de salud que contribuyen al diagnóstico tardío de la lepra en un área endémica del Nordeste de Brasil. Se trata de un estudio transversal que incluyó a 120 pacientes con lepra. Se obtuvieron datos demográficos, clínicos e informaciones sobre los factores relacionados con el individuo y el sistema de salud que contribuyen al diagnóstico tardío de la lepra. Se estimó el retraso del diagnóstico en meses para cada participante a través de entrevistas. Se realizó un análisis multivariante por regresión de Poisson entre el resultado y las variables independientes. La mediana de retraso en el diagnóstico de lepra fue de 10,5 (4,0-24,0) meses. Aproximadamente el 12,6% de los participantes tenían grado de discapacidad física 2 (GIF 2) en el momento del diagnóstico. En el análisis multivariante por regresión de Poisson se encontró que los hombres, ancianos, la baja escolaridad, vivir en área urbana, la lepra multibacilar, la lepra tuberculoide, no buscar atención médica inmediatamente tras notar los primeros síntomas, la sospecha de lepra, las derivaciones excesivas y la necesidad de tres o más consultas para confirmar el diagnóstico se asociaron con un mayor retraso del diagnóstico. Este estudio encontró un retraso significativo en el diagnóstico de la lepra en Arapiraca, Nordeste de Brasil, lo que puede explicar la tasa continuamente alta de GIF 2 entre los nuevos casos. Factores relacionados con el individuo y el sistema de salud se asociaron con el mayor retraso del diagnóstico. Intervenciones para aumentar la concienciación sobre la enfermedad entre la población general y fortalecer la atención primaria de salud son urgentemente necesarias.

## Introduction

Leprosy, also known as Hansen’s disease, is a chronic infectious disease caused by the bacterium *Mycobacterium leprae*, which mainly affects the skin and nerves ^1^ ^,^ ^2^. Despite being curable, leprosy remains a major public health problem in many countries, including Brazil ^3^. One of the main challenges in controlling leprosy is the delay in diagnosis, which can lead to severe physical and psychological consequences for patients ^4^ ^,^ ^5^ ^,^ ^6^ ^,^ ^7^ ^,^ ^8^.

This delay in diagnosis has been attributed to several factors, including lack of knowledge and awareness among healthcare providers ^9^, social stigma and discrimination associated with the disease, and factors related to the community and patients ^10^. However, few studies concomitantly assess factors related to the individual and the health system.

Identifying the factors that contribute to delayed diagnosis of leprosy is crucial for developing effective strategies to improve early detection and control of the disease. In Brazil, leprosy is endemic in several regions and, as in other low-endemic countries, has a highly clustered pattern of occurrence at the sub-national level, with areas in the North, Central-West, and Northeast bearing the greatest burden of the disease ^11^. Due to this variation, studies should be conducted in these regions to identify the factors that contribute to delayed diagnosis in these areas and inform policymakers and program managers to develop targeted interventions to improve early detection and leprosy control.

This study investigated the factors related to the individual and the health system that contribute to delayed diagnosis of leprosy in an endemic area for the infection in Northeastern Brazil.

## Methods

### Study design and participants

This is a cross-sectional study of individuals with leprosy aged ≥ 15 years diagnosed from 2015 to 2022 in Arapiraca (Alagoas State, Northeastern Brazil). Arapiraca has a population of ~235,000 inhabitants and hosts the second health macroregion of the state of Alagoas, which is a reference for the care of an estimated 1.1 million individuals. In 2021, the rate of new leprosy cases in Arapiraca was 10.7 per 100,000 inhabitants, and the rate of grade 2 disability (G2D) among new cases of leprosy was 20%, which is considered high endemicity by the parameters of the Brazilian Ministry of Health.

All consecutive patients who visited the leprosy and tuberculosis sector of the Integrated Reference Center of Arapiraca from November 2021 to June 2022, who were receiving multidrug therapy or who had been previously treated for leprosy and were returning to the center for follow-up, were invited to participate. Individuals with cognitive deficits, who did not understand the questions (self-report), or who did not have complete clinical data were excluded. Individuals who were diagnosed before 2015 were also excluded to minimize the risk of memory bias, the threshold for which was identified in the pilot study.

### Sample size

A sample of 118 individuals with leprosy was required to determine the factors that contribute to delayed diagnosis. This sample size was estimated based on the number of patients registered at the reference center from 2015 and 2022 (N = 170), assuming that 50% of individuals would have delayed diagnosis of leprosy, considering a 5% type I error (α) and a 95% confidence interval (95%CI).

### Questionnaires and procedures

After obtaining written consent, participants were interviewed using a structured questionnaire based on the study by Gómez et al. ^12^ to obtain demographic and clinical information, factors that may contribute to delayed diagnosis of leprosy. They were asked about the date (month/year) of the onset of signs and symptoms, the time from symptom onset to seeking health services, and the time from seeking health services to the definitive diagnosis of leprosy.

Sociodemographic and clinical data included: sex, age, schooling (in years), area of residence (urban or rural area), operational classification (paucibacillary [PB] or multibacillary [MB]), clinical form, degree of disability, and leprosy reaction (absent or present). Participants were classified as PB leprosy if they had ≤ 5 skin lesions, only one affected nerve trunk, or both; or negative smear findings; and as MB leprosy if they had > 5 skin lesions, more than one affected nerve trunk, or both; or positive smear findings ^13^. The clinical forms included indeterminate, tuberculoid, borderline, and lepromatous presentations ^14^. The degree of disability was based on the World Health Organization (WHO) disability grading, where grade 0 (G0D) represents no disability; grade 1 (G1D), a decrease or loss of sensation in the eyes, hands, and/or feet without visible deformity; and G2D, loss of sensation and visible deformities ^15^. Nerves were considered to be affected in the presence of pain or nerve thickening on palpation, loss of sensation with the monofilament test or motor impairment ^15^. Leprosy reactions included episodes characterized by acute inflammation of skin lesions or nerves (type 1) and the appearance of inflamed skin nodules with or without neuritis (type 2) ^16^. Participants’ medical records and the notification forms in the Brazilian Information System for Notificable Diseases (SINAN, acronym in Portuguese) were reviewed to confirm the clinical information.

All participants were interviewed face-to-face in a quiet and private place. A trained member of the research team conducted the interviews, and the research assistant was not involved in the treatment of the participants included.

### Outcome

The outcome variable was the time elapsed from the onset of signs and symptoms to diagnosis (in months) and is referred to as diagnostic delay in this study.

### Data analysis

Categorical variables were described using frequencies and percentages. The assumptions of normality were assessed using the Shapiro-Wilk test and homoscedasticity by the Levene’s test. Nonparametric tests were used to verify the significance of the distributions between the study variables, as most of the dataset had a skewed distribution. To determine the association between the factors related to delayed diagnosis, a multivariate Poisson’s regression analysis was performed between the outcome variable (delay in months) and the independent variables, except for the degree of disability, since it has been used as a proxy for diagnostic delay. P-values < 5% were statistically significant. All analyses were performed using Stata, version 14.0 (https://www.stata.com).

### Ethical approval

The study was approved by the Human Research Ethics Committee of the Federal University of Sergipe (CAAE 47713121.9.0000.5546, protocol n. 5,061,479) and conducted in accordance with the *Declaration of Helsinki*. All participants gave their written informed consent. In the case of minors, the parents or guardians provided written informed consent and the minors signed an assent form.

## Results

A total of 125 individuals with leprosy were invited to participate in the study. However, five did not have complete clinical data in their medical reports and were excluded. Therefore, the final sample size was 120 participants.

Of the 120 participants included, 61 (50.8%) were men and 59 (49.2%) were women. The median (interquartile range - IQR) age was 45 (34.0-56.0) years and the 40-49 age group was the most prevalent (30; 25%). The median (IQR) of schooling was 5 (2.5-10.0) years and 55 (45.9%) participants had 0 to 4 years of schooling. In total, 74 (61.8%) participants were mixed-race, 24 (20%) were white, and 21 (17.5%) were black. Most participants lived in urban areas (91; 75.8%). Regarding the degree of disability, 66 (55%) had G0D, 39 (32.5%) had G1D, and 15 (12.5%) had G2D. Regarding the operational classification at diagnosis, 92 (76.7%) had MB leprosy. Regarding the clinical form, 24 (20%) were classified as having tuberculoid leprosy, 63 (52.5%) had borderline leprosy, and 29 (24.2%) had lepromatous leprosy. A total of 106 individuals with leprosy underwent smear microscopy, of which 33 (27.5%) tested positive. Moreover, 41 (34.1%) individuals had leprosy reactions, of which 22 (18.3%) had type 1 reaction (reversal reaction), 16 (13.3%) had type 2 reaction (erythema nodosum leprosum [ENL]), and three (2.5%) had a mixed reaction (type 1 and type 2) (Table 1).

**Table 1:** Demographic and clinical characteristics of individuals with leprosy treated at the municipality of Arapiraca, Alagoas State, Brazil, from 2015 to 2022.

Characteristics	n (%)
Sex	
Male	61 (50.8)
Female	59 (49.2)
Age group (years)	
15-19	8 (6.7)
20-29	12 (10.0)
30-39	24 (20.0)
40-49	30 (25.0)
50-59	20 (16.7)
≥ 60	26 (21.7)
Ethnicity/Skin color	
White	24 (20.0)
Black	21 (17.5)
Mixed-race	74 (61.8)
Asian	1 (0.8)
Schooling (years)	
0-4	55 (45.9)
5-8	25 (20.8)
≥ 9	40 (33.3)
Area of residence	
Urban	91 (75.8)
Rural	29 (24.2)
Degree of disability at diagnosis	
Grade 0	66 (55.0)
Grade 1	39 (32.5)
Grade 2	15 (12.5)
Operational classification	
Paucibacillary	28 (23.3)
Multibacillary	92 (76.7)
Clinic form	
Indeterminate	4 (3.3)
Tuberculoid	24 (20.0)
Borderline	63 (52.5)
Lepromatous	29 (24.2)
Smear microscopy	
Negative	73 (60.8)
Positive	33 (27.5)
Not performed	14 (11.7)
Leprosy reaction	
Type 1	22 (18.3)
Type 2	16 (13.3)
Type 1 and 2	3 (2.5)

The median (IQR) time from the onset of signs and symptoms to seeking healthcare was 3.0 (1.0-7.5) months. The median (IQR) time from the onset of signs and symptoms and the definitive diagnosis of leprosy was 10.5 (4.0-24.0) months.

Table 2 shows the factors related to the individual that contribute to delayed diagnosis. In this study, 65 (54.2%) participants did not seek healthcare facilities immediately after noticing the first signs and symptoms, of whom 54 (83.1%) thought that their signs and symptoms were not important, two (3.1%) reported lack of money, and two (3.1%) were afraid of being diagnosed with a serious illness. In total, 109 (90.8%) individuals had never suspected that they had leprosy.

**Table 2:** Potential delay factors related to the individual with leprosy in the municipality of Arapiraca, Alagoas State, Brazil, from 2015 to 2022.

Factors	n (%)
Seeking healthcare immediately after noticing the first symptoms	
Yes	55 (45.8)
No	65 (54.2)
Reasons for not seeking healthcare immediately after noticing the first symptoms	
Did not think the symptoms were important enough to seek care	54 (45.0)
Lack of money	2 (1.7)
Fear of being affected by some serious illness	2 (1.7)
Other	7 (5.8)
The participant suspected leprosy before diagnosis by a health professional	
Yes	11 (9.2)
No	109 (90.8)

Table 3 shows the potential delay factors related to the health system. Most participants (116; 96.7%) were treated at the first health service they sought. However, 31 (25.8%) were misdiagnosed and mistreated for other medical conditions, with dermatological conditions (71%) being the most common misdiagnosis. In total, 114 (95%) were referred from primary health care (PHC) to a specialized healthcare service, of which 109 (93.9%) were referred to the leprosy reference center. Of the participants, 34 (28.3%) had one consultation, 51 (42.5%) had two, 26 (21.7%) had three, and nine (7.5%) had more than four consultations to establish the diagnosis of leprosy. Dermatologists and PHC physicians were the professionals who suspected leprosy the most, with 40 (33.9%) and 37 (31.4%), respectively. However, the diagnosis was mostly confirmed by a leprologist (95; 79.2%) or a dermatologist (20; 16.7%), respectively.

**Table 3:** Potential factors related to the health service and professionals according to individuals with leprosy in the municipality of Arapiraca, Alagoas State, Brazil, from 2015 to 2022.

Factors	n (%)
The participant was treated at the first health service sought	
Yes	116 (96.7)
No	4 (3.3)
Reason for not receiving healthcare	
Lack of a physician	4 (3.3)
Initial misdiagnosis and mistreatment	
Yes	31 (25.8)
No	89 (74.2)
Misdiagnosed conditions	
Dermatological conditions *	22 (71.0)
Rheumatological conditions **	2 (6.4)
Orthopedic conditions ***	2 (6.4)
Other neglected tropical disease ^#^	1 (3.2)
Did not know	4 (13.0)
Received a diagnosis of leprosy at the first consultation	
Yes	28 (23.3)
No	92 (76.7)
Number of health services attended before the diagnosis of leprosy	
1	65 (54.2)
2	38 (31.7)
3	4 (3.3)
4 or more	13 (10.8)
Referred to a specialist	
Yes	114 (95.0)
No	6 (5.0)
Specialist referred to	
Leprosy referral center	109 (93.9)
Medical specialties center	2 (1.7)
Dermatologist	2 (1.7)
Allergy specialist	1 (0.8)
Number of consultations required to receive the diagnosis of leprosy	
1	34 (28.3)
2	51 (42.5)
3	26 (21.7)
4 or more	9 (7.5)
Health professional who suspected leprosy	
Dermatologist	41 (34.2)
FHS physician	37 (30.9)
FHS nurse	19 (15.8)
Leprologist	10 (8.3)
Community health professional	3 (2.5)
Other medical specialty	10 (8.3)
Health professional who confirmed the diagnosis of leprosy	
Leprologist	95 (79.2)
Dermatologist	20 (16.7)
FHS physician	4 (3.3)
Allergy specialist	1 (0.8)

FHS: Family Health Strategy.

* Atopic dermatitis: 11; fungal infection: 8; vasculitis: 1; pityriasis: 1; chronic urticaria: 1;

** Rheumatoid arthritis: 1; lupus: 1;

*** Achilles tendon enthesopathy: 2;

^#^ Syphilis: 1.

Bivariate analysis showed that men had a higher risk of delayed diagnosis compared with women (incidence risk ratio - IRR = 1.28; 95%CI: 1.20-1.40; p < 0.001). Individuals aged 30-39 years had a 1.22-fold increased risk of delayed diagnosis compared with the 15-19 age group. Mixed-race individuals (IRR = 1.21; 95%CI: 1.10-1.37; p < 0.001) had a higher risk of delayed diagnosis compared with white individuals; participants living in urban areas (IRR = 1.68; 95%CI: 1.49-1.87; p < 0.001); and MB leprosy (IRR = 1.56; 95%CI: 1.40-1.74; p < 0.001). Tuberculoid, borderline, and lepromatous leprosy had a higher risk of delayed diagnosis compared with the indeterminate form (Table 4).

**Table 4:** Multivariate Poisson’s regression with the factors associated with the time from symptom onset to diagnosis of leprosy in the municipality of Arapiraca, Alagoas State, Brazil, from 2015 to 2022.

Characteristics	Bivariate analysis			Multivariate analysis (pseudo R^2^ = 0.49)		
	IRR	95%CI	p-value	Adjusted IRR	95%CI	p-value
Sex						
Female	Reference			Reference	Reference	Reference
Male	1.28	1.20-1.40	< 0.001	1.27	1.13-1.43	< 0.001
Age group (years)						
15-19	Reference	Reference	Reference	Reference	Reference	Reference
20-29	0.73	0.59-0.90	0.004	0.61	0.46-0.81	0.001
30-39	1.22	1.03-1.45	0.02	1.90	1.51-2.38	< 0.001
40-49	0.89	0.75-1.06	0.216	2.24	1.77-2.84	< 0.001
50-59	0.74	0.62-0.89	0.002	2.15	1.63-2.82	< 0.001
≥ 60	0.84	0.71-1.00	0.064	2.93	2.20-3.90	< 0.001
Ethnicity/Skin color						
White	Reference	Reference	Reference	Reference	Reference	Reference
Black	0.85	0.73-0.98	0.03	0.82	0.68-0.97	0.011
Mixed-race	1.21	1.10-1.35	< 0.001	0.75	0.69-0.89	< 0.001
Asian	1.38	0.91-2.08	0.124	1.09	0.60-1.80	0.712
Schooling (years)						
0-4	0.78	0.71-0.86	< 0.001	0.68	0.58-0.81	< 0.001
5-8	1.13	1.02-1.26	0.021	1.35	1.16-1.57	< 0.001
≥ 9	Reference	Reference	Reference	Reference	Reference	Reference
Area of residence						
Rural	Reference	Reference	Reference	Reference	Reference	Reference
Urban	1.68	1.49-1.87	< 0.001	1.33	1.16-1.52	< 0.001
WHO operational classification						
Paucibacillary	Reference	Reference	Reference	Reference	Reference	Reference
Multibacillary	1.56	1.40-1.74	< 0.001	7.60	4.65-12.42	< 0.001
Clinical form						
Indeterminate	Reference	Reference	Reference	Reference	Reference	Reference
Tuberculoid	3.27	2.06-5.19	< 0.001	3.61	2.21-6.29	< 0.001
Borderline	3.71	2.36-5.85	< 0.001	1.06	0.50-2.36	0.878
Lepromatous	6.10	3.86-9.59	< 0.001	1.94	0.86-4.40	0.112
Seeking healthcare immediately after noticing the first symptoms						
Yes	0.68	0.62-0.74	< 0.001	0.44	0.39-0.49	< 0.001
The participant was treated at the first health service sought						
Yes	0.61	0.51-0.73	< 0.001	0.48	0.37-0.58	< 0.001
The participant suspected leprosy before diagnosis by a health professional						
Yes	1.41	1.25-1.60	< 0.001	1.51	1.24-1.83	< 0.001
Health professional who suspected leprosy						
FHS physician	0.71	0.61-0.82	< 0.001	0.88	0.73-1.05	0.172
FHS nurse	1.01	0.86-1.18	0.910	1.90	1.54-2.33	< 0.001
Leprologist	0.61	0.48-0.75	< 0.001	0.90	0.68-0.98	< 0.001
Dermatologist	1.04	0.90-1.20	0.563	1.43	1.15-1.78	0.001
Other	Reference	Reference	Reference	Reference	Reference	Reference
Number of health services attended before the diagnosis of leprosy						
1	Reference	Reference	Reference	Reference	Reference	Reference
2	1.48	1.35-1.62	< 0.001	0.98	0.85-1.14	0.857
3	0.51	0.36-0.72	< 0.001	1.15	1.44-2.35	< 0.001
4 or more	1.92	1.70-2.16	< 0.001	1.77	1.40-2.24	< 0.001
Number of consultations required to receive the diagnosis of leprosy						
1	Reference	Reference	Reference	Reference	Reference	Reference
2	1.18	1.06-1.31	0.002	1.03	0.90-1.17	0.668
3	1.12	0.98-1.27	0.080	1.25	1.06-1.48	0.008
4 or more	3.10	2.73-3.53	< 0.001	5.46	4.51-6.61	< 0.001
Initial misdiagnosis and mistreatment						
Yes	1.29	1.18-1.41	< 0.001	1.56	1.32-1.82	< 0.001
Health professional who confirmed the diagnosis of leprosy						
FHS physician	Reference	Reference	Reference	Reference	Reference	Reference
Leprologist	1.69	1.25-2.28	0.001	1.34	0.96-1.88	0.083
Dermatologist	2.23	1.64-3.04	< 0.001	1.35	0.95-1.92	0.084

95%CI: 95% confidence interval; FHS: Family Health Strategy; IRR: incidence risk ratio; WHO: World Health Organization.

The bivariate analysis also showed that participants who sought healthcare facilities immediately after noticing their symptoms (IRR = 0.68; 95%CI: 0.62-0.74; p < 0.001) and individuals who obtained care at the first service they sought (IRR = 0.61; 95%CI: 0.51-0.73; p < 0.001) had a reduction in the time to diagnosis of leprosy. On the other hand, participants who suspected that they had leprosy had a delay in diagnosis 1.41 times higher than those who did not. The risk of delayed diagnosis increased with the raise in the number of health services visited and consultations required to diagnose leprosy (Table 4).

The multivariate Poisson’s regression found that men had a 1.27 times higher risk of delayed diagnosis compared with women. The risk of delayed diagnosis increased with increasing age, as individuals aged ≥ 60 years had a 2.93 times higher risk of delayed diagnosis compared with the 15-19 age group. Mixed-race participants had a shorter time to diagnosis compared with white individuals. Participants living in urban areas had an increased risk of delayed diagnosis (IRR = 1.33; 95%CI: 1.16-1.52; p < 0.001). MB leprosy had a 7.6 times higher risk of delayed diagnosis than PB leprosy. Tuberculoid leprosy had a 3.6 times higher risk of delayed diagnosis compared with the indeterminate form (Table 4).

Participants who sought healthcare facilities immediately after noticing their signs and symptoms (IRR = 0.44; 95%CI: 0.39-0.49; p < 0.001) and individuals who obtained care at the first service they sought (IRR = 0.48; 95%CI: 0.37-0.58; p < 0.001) had a shorter time to diagnosis of leprosy. Individuals who suspected that they had leprosy had a 1.51-fold delay in diagnosis compared with those who did not. The risk of delayed diagnosis increased with the increasing number of health services visited and consultations required to diagnose leprosy (Table 4).

## Discussion

We report a median time of three months for individuals with leprosy to seek care from health services; and a median time of 10 months from the onset of signs and symptoms to the definitive diagnosis of leprosy. These values are similar to another Brazilian study conducted in the state of Espírito Santo ^17^, but substantially lower than the median reported in other countries ^12^ ^,^ ^18^. Our findings highlight that the main individual-related factors that contribute to delayed diagnosis were sex, older age, residing in urban areas, not seeking a healthcare facility immediately after noticing the first signs and symptoms, and the patient’s suspicion of leprosy. The factors related to the health system that contribute to delayed diagnosis of leprosy included the lack of immediate care at the first health service sought, especially when access to a physician was unavailable, excessive referrals, and the need for three or more consultations to confirm the diagnosis.

Men were more likely to have delayed diagnosis of leprosy than women, in line with other studies ^19^ ^,^ ^20^. Men are often reluctant to seek healthcare and neglect signs and symptoms, especially when they are mild, such as initial signs and symptoms of leprosy ^18^ ^,^ ^20^ ^,^ ^21^. Health professionals should be aware of the increased risk of delayed diagnosis and physical disability in men ^7^ (a proxy for delayed diagnosis) during active case finding and contact tracing, to ensure that male contacts and secondary cases are not missed.

The risk of delayed leprosy diagnosis increased with age and our findings were consistent with other studies ^10^ ^,^ ^22^ ^,^ ^23^ ^,^ ^24^. This can be confounded by the long incubation period of leprosy, which can range from several months to years ^1^ ^,^ ^2^ ^,^ ^4^. Moreover, as age advances, individuals are more likely to have a poorer health status, and physicians may suspect other diseases that are more prevalent in each age group.

Although most of the data on delayed diagnosis of leprosy include living in rural areas as a risk factor ^10^ ^,^ ^18^ ^,^ ^25^, in this study, living in urban areas was associated with longer diagnostic delay. Notably, the classification of urban/rural areas in several Brazilian regions is imprecise, especially regarding small- and medium-sized municipalities. Moreover, Brazil is a country with a significant rural-urban migration flow, which has intensified over the past few decades. This phenomenon is primarily driven by factors such as the search for employment opportunities and access to education and healthcare ^26^ ^,^ ^27^.

Not seeking a healthcare center immediately after noticing the first signs and symptoms of leprosy was a significant factor associated with delayed diagnosis. Despite living in a leprosy endemic area, many individuals may not recognize the signs and symptoms of leprosy or seek medical care until the symptoms become severe ^12^ ^,^ ^20^. Paradoxically, individuals who suspected leprosy were more likely to have delayed diagnosis. Leprosy has historically been associated with social exclusion and discrimination, with reports suggesting that patients may hide their symptoms and delay seeking medical care ^10^ ^,^ ^12^. This has the potential to further exacerbate the health consequences of leprosy. To address these issues, we recommend that efforts be made to raise awareness and educate the community about leprosy, to combat stigma and improve access to healthcare services.

In this study, participants with the tuberculoid form had a higher risk of delayed diagnosis compared with participants with the indeterminate form. However, the multivariate analysis found no other association concerning the borderline and lepromatous clinical forms.

PB leprosy is the main challenge for the diagnosis of leprosy in clinical practice. Thus, our findings on the increased risk of MB leprosy for late diagnosis may be interpreted as a marker of delay. Patients may have sought health care when they still had few and undefined skin lesions and, given the difficulty of physicians in establishing an early diagnosis, this was postponed. This may partly explain the excessive number of referrals and consultations, with significant consequences for the time between the first signs and symptoms and the definitive diagnosis. This reinforces the importance of strengthening the capacity of diagnosing leprosy in PHC and the continuous training of healthcare professionals to better meet the demands of the population with greater resolution, avoiding unnecessary referrals.

Moreover, the high number of referrals to specialized services and consultations to confirm leprosy is an important risk factor for the increase in the time to diagnosis of leprosy ^9^ ^,^ ^10^ ^,^ ^12^ ^,^ ^28^. This reinforces the importance of continuous training on the diagnosis and treatment of leprosy, especially in countries where the disease is still a public health problem; and the need for greater investment in the development of highly accurate diagnostic tests for leprosy that can be used in PHC units ^4^.

Since 2004, the Brazilian National Leprosy Control Program (PNCH, acronym in Portuguese), which aims to expand and facilitate the early diagnosis and treatment of leprosy and, consequently, reduce leprosy-related physical disabilities in Brazil, has been adopting the strategy of decentralizing and strengthening PHC, the central pillar of leprosy control ^29^. However, healthcare professionals are still reluctant to decentralize and a high proportion of patients are referred to specialized leprosy centers for diagnostic confirmation ^30^. This further exacerbates the pressure on specialized centers, since their original purpose lies on caring for and managing the most complex cases, rather than those that can be effectively treated in PHC ^29^.

A quarter of the participants in our study were misdiagnosed and mistreated. This shows that healthcare professionals may not consider leprosy as a possible diagnosis, even in areas endemic to leprosy. Organizing greater awareness is necessary ^31^. However, this is confounded by the clinical presentation of leprosy, which can be variable and often mimics other medical conditions ^12^ ^,^ ^31^. Studies conducted in Colombia ^12^, the United Kingdom ^31^, India ^32^, and other regions of Brazil ^20^ also found a high proportion of individuals being misdiagnosed and mistreated before confirmation of leprosy.

Timely diagnosis of leprosy and early treatment are important to break the transmission chain ^9^ ^,^ ^10^ ^,^ ^12^. Without early intervention, leprosy can cause irreversible nerve damage, impairment, and disability, making it not only a health issue but also a social problem, as stigma and discrimination against affected individuals persist ^6^ ^,^ ^7^ ^,^ ^8^. By diagnosing and treating leprosy early, we not only prevent the suffering and disabilities of those affected, but also reduce the potential for further transmission within communities ^1^ ^,^ ^3^.

This study has some limitations. It relied on the recollection of participants, which would introduce a recall bias inherent in studies like this, despite the preventive measures adopted in data collection. This study was performed with patients with leprosy from a leprosy reference center and our findings may not be readily extrapolated to individuals treated in PHC units. Despite this, this study shows the reality of several similar configurations that can be found in endemic areas with reference centers, which persist as the main sites for leprosy diagnosis.

In conclusion, multiple factors related to the individual and the health system cause delayed diagnosis of leprosy. Addressing these factors will require a coordinated effort among health professionals, governments, and other stakeholders to raise awareness and strengthen healthcare systems to ensure timely diagnosis and treatment of leprosy, especially in endemic areas such as Brazil.
